# Supplementary material for: The Deinococcus protease PprI senses DNA damage by directly interacting with single-stranded DNA
Source: Nat Commun. 2024 Feb 29;15:1892. doi: 10.1038/s41467-024-46208-9 (PMC10904395; doi:10.1038/s41467-024-46208-9)
Supplement: Supplementary file 1 — supplementary information [file 41467_2024_46208_MOESM1_ESM.pdf]

The *Deinococcus* protease PprI senses DNA damage by directly interacting with single-stranded DNA

Huizhi Lu[1,5], Zijing Chen[1,5], Teng Xie[1,2], Shitong Zhong[1], Shasha Suo[1], Shuang Song[1], Liangyan Wang[1], Hong Xu[1,3], Bing Tian[1,3], Ye Zhao[1,3]*, Ruhong Zhou[1,2,3,4]* & Yuejin Hua[1,3]*

1. MOE Key Laboratory of Biosystems Homeostasis & Protection, Institute of Biophysics, College of Life Sciences, Zhejiang University, China

2. Shanghai Institute for Advanced Study, Zhejiang University, Shanghai, China

3. Cancer Center, Zhejiang University, Hangzhou, Zhejiang, China

4. Department of Chemistry, Columbia University, New York, United States

5. These authors contributed equally to this work

* To whom correspondence should be addressed. Tel: 86-571-86971703; Fax: 86-571-86971703; Email: H. Y. (yjhua@zju.edu.cn)

Correspondence may also be addressed to yezhao@zju.edu.cn or rhzhou@zju.edu.cn

Keywords: DNA damage response, single-stranded DNA, activation, dimer, protease, *Deinococcus*

Supplementary Table 1: Data collection and refinement statistics

PDB ID	PprI apo<br>8SLM	PprI-ssDNA<br>8SLN
**Data collection**		
Space group	P 63 22	P 31 2 1
Cell dimensions		
a, b, c (Å)	120.77 120.77 100.66	84.99, 84.99, 88.55
	90 90 120	90, 90, 120
Wavelength (Å)	0.9792	0.9792
Resolution (Å)	30-2.8	30-2.2
Rsym (%)	4.1 (54.1)	3.5 (48.4)
I/σI	21.4 (2.6)	22.2 (3.0)
Completeness (%)	96.9 (99.4)	99.7 (99.5)
Redundancy	4.1 (4.2)	5.5 (5.5)
**Refinement**		
Resolution (Å)	30-2.8	30-2.2
No. reflections	10704	19049
Rwork/ Rfree	24.7/28.4	23.3/26.5
No. atoms		
Protein/DNA	1838/-	1825/224
ion	6	1
B-factors		
Protein/DNA	85.1/-	76.5/121.7
ion	144.9	56.9
R.m.s deviations		
Bond lengths (Å)	0.012	0.004
Bond angles (º)	1.40	0.67
Ramachandran statistics		
Favored (%)	97.93	97.88
Allowed (%)	2.07	2.12
Outliers (%)	0	0

Statistics for the highest-resolution shell are shown in parentheses.

Supplementary Table 2: Strains and plasmids

Strain and plasmid	Description	Source
**Strains**		
E. coli		
Trans5α	*E. coli* cloning strain	Transgen
BL21 (DE3)	*E. coli* expression strain	Transgen
BL21-28-PprI	*E. coli* expression strain for PprI protein	This study
BL21-HMT-PprI	*E. coli* expression strain for PprI protein with removable tag	This study
BL21-28-PprI NM	*E. coli* expression strain for PprI-NM protein	This study
BL21-28-PprI Patch1	*E. coli* expression strain for PprI-patch1 (R85A/R207A/R267A)	This study
BL21-28-PprI Patch2	*E. coli* expression strain for PprI-patch2 (L22A/K26A/R117A)	This study
BL21-28-PprI Patch3	*E. coli* expression strain for PprI-patch3 (R220A/R250A/S251A)	This study
BL21-28-PprI 2mut	*E. coli* expression strain for PprI-2mut (H46A/F58A)	This study
BL21-28-PprI 4mut	*E. coli* expression strain for PprI-4mut (D69A/E71A/H72A/R73A)	This study
BL21-28-PprI 6mut	*E. coli* expression strain for PprI-6mut (H46A/F58A/D69A/E71A/H72A/R73A)	This study
BL21-28-DdrO E116A	*E. coli* expression strain for DdrO (E116A)	This study
BL21-28-DdrO L117A	*E. coli* expression strain for DdrO (L117A)	This study
BL21-28-DdrO R118A	*E. coli* expression strain for DdrO (R118A)	This study
BL21-28-DdrO G119A	*E. coli* expression strain for DdrO (G119A)	This study
D. radiodurans		
wild-type R1	ATCC 13939	Lab stock
YR1	As R1 but *pprI::kana*	Lab stock
YR1-*dg_pprI*	As YR1 but compensated with pk-*dgpprI*	This study
YR1-NM	As YR1 but compensated with pk-*dgpprIΔC*	This study
YR1-patch1	As YR1 but compensated with pk-*dgpprIpatch1*	This study
YR1-patch2	As YR1 but compensated with pk-*dgpprIpatch2*	This study
YR1-patch3	As YR1 but compensated with pk-*dgpprIpatch3*	This study
YR1-2mut	As YR1 but compensated with pk-*dgpprI2mut*	This study
YR1-4mut	As YR1 but compensated with pk-*dgpprI4mut*	This study
YR1-6mut	As YR1 but compensated with pk-*dgpprI6mut*	This study
D. geothermalis		
wild-type strain	DSM11300	Lab stock
**Plasmids**		
pMD18-T vector	For TA cloning	Lab stock
pET28a	For protein expression	Lab stock
pET28-HMT	Modified from pET28a containing 6×His-tag, maltose binding protein and TEV protease site	Lab stock
28a-*dgddrO*	As pET28a but ligated with *dgddrO*	Lab stock
28a-*dgpprI*	As pET28a but ligated with *dgpprI*	Lab stock

**Strain and plasmid**	**Description**	**Source**
HMT-*dgpprI*	As pET28-HMT but ligated with dgpprI	This study
28a-*dgpprIΔC*	As pET28a but ligated with *dgpprIΔC*	This study
28a-*dgpprIR85A*	As pET28a but ligated with *dgpprIR85A*	This study
28a-*dgpprIR207A*	As pET28a but ligated with *dgpprIR207A*	This study
28a-*dgpprIR267A*	As pET28a but ligated with *dgpprIR267A*	This study
28a-*dgpprIpatch1*	As pET28a but ligated with *dgpprIR85/207/267A*	This study
28a-*dgpprIL22*A	As pET28a but ligated with *dgpprIL22A*	This study
28a-*dgpprIK26A*	As pET28a but ligated with *dgpprIK26A*	This study
28a-*dgpprIR117A*	As pET28a but ligated with *dgpprIR267A*	This study
28a-*dgpprIpatch2*	As pET28a but ligated with *dgpprIL22/K26/R117A*	This study
28a-*dgpprIR220A*	As pET28a but ligated with *dgpprIR220A*	This study
28a-*dgpprIR250A*	As pET28a but ligated with *dgpprIR250A*	This study
28a-*dgpprIS251A*	As pET28a but ligated with *dgpprIS251A*	This study
28a-*dgpprIpatch3*	As pET28a but ligated with *dgpprIR220/R250/S251A*	This study
28a-*dgpprI2mut*	As pET28a but ligated with *dgpprIH46A/F58A*	This study
28a-*dgpprI4mut*	As pET28a but ligated with *dgpprID69A/E71A/H72A/R73A*	This study
28a-*dgpprI6mut*	As pET28a but ligated with *dgpprI H46A/F58A/D69A/E71A/H72A/R73A*	This study
28a-*dgpprI-eCFP*	As pET28a but ligated with *dgpprI* fused with *eCFP*	This study
28a-*dgpprI-eYFP*	As pET28a but ligated with *dgpprI* fused with *eYFP*	This study
28a-*dgddrO*-eCFP	As pET28a but ligated with *dgddrO* fused with eCFP	This study
28a-*dgddrOmut*-eCFP	As pET28a but ligated with *dgddrOR118A* fused with eCFP	This study
28a-*dgddrOE116A*	As pET28a but ligated with *dgddrOE116A*	This study
28a-*dgddrOL117A*	As pET28a but ligated with *dgddrOL117A*	This study
28a-*dgddrOR118A*	As pET28a but ligated with *dgddrOR118A*	This study
28a-*dgddrOG119A*	As pET28a but ligated with *dgddrOG119A*	This study
pRADK	*E. coli-D. radiodurans* shuttle vector	Lab stock
pk-*dgpprI*	As pRADK but *kan^r* was replaced with *dgpprI* gene	This study
pk-*dgpprINM*	As pRADK but *kan^r* was replaced with *dgpprIΔC* fragment	This study
pk-*dgpprIpatch1*	As pRADK but *kan^r* was replaced with *dgpprIpatch1* mutation fragment	This study
pk-*dgpprIpatch2*	As pRADK but *kan^r* was replaced with *dgpprIpatch2* mutation fragment	This study
pk-*dgpprIpatch3*	As pRADK but *kan^r* was replaced with *dgpprIpatch3* mutation fragment	This study
pk-*dgpprI2mut*	As pRADK but *kan^r* was replaced with *dgpprI2mut* fragment	This study
pk-*dgpprI4mut*	As pRADK but *kan^r* was replaced with *dgpprI4mut* fragment	This study
pk-*dgpprI6mut*	As pRADK but *kan^r* was replaced with *dgpprI6mut* fragment	This study

Supplementary Table 3: Primers, DNA and RNA substrates

	Sequence (5′→3′)	Label
**Co-crystallization**		
29nt	TCATGAGCAGTTTTTTTTTTTTTTTTTT	None
**EMSA assays**		
35nt RNA	CGCUCUUCGCCAUUCUCUUGAAGUUUCAAACUUGG	5'FAM
35 bp F	CGCTCTTCGCCATTCTCTTGAAGTTTCAAACTTGG	5'FAM
35 bp R	CCAAGTTTGAAACTTCAAGAGAATGGCGAAGAGCG	None
35nt	CGCTCTTCGCCATTCTCTTGAAGTTTCAAACTTGG	5'FAM
40nt	GTTATCACAGGTTATCTAGAGGTAGTAGAGTCAGAGGTAG	5'FAM
30nt	GTTATCACAGGTTATCTAGAGGTAGTAGAG	5'FAM
28nt	GTTATCACAGGTTATCAGAGGTATAGAG	5'FAM
26nt	GTTATCACAGGTATCAGAGTATAGAG	5'FAM
24nt	GTTATCACAGGTATCAGAGTAGAG	5'FAM
22nt	GTTATCACAGGTATCAGTAGAG	5'FAM
20nt	GTTATCACAGGTTATCAGAG	5'FAM
18nt	GTTATCACAGGTACAGAG	5'FAM
16nt	GTTATCACAGTCAGAG	5'FAM
14nt	GTATCAGAGCAGTG	5'FAM
12nt	GTATCAAGAGTG	5'FAM
10nt	GTTATCAGAG	5'FAM
9nt	GTTACAGAG	5'FAM
8nt	GTTCAGAG	5'FAM
7nt	GTCAGAG	5'FAM
6nt	GTCAAG	5'FAM
5nt	GTCAG	5'FAM
**Clone and site mutation**		
dg_pprI F	GTGCCGCGCGGCAGCCATATGACGCAGGGCCAGACC	
dg_pprI R	ACGGAGCTCGAATTCGGATCCTCAGACACCCGACTCATCCTG	
dg_pprIΔC R	ACGGAGCTCGAATTCGGATCCTCAGGTTCGCTCCGCCAG	
prad-*dg_pprI* F	CTCACAGGAGGACCCCATATGGTGACGCAGGGCCAGACC	
prad-*dg_pprI* R	CCTGCAGGTCGAATCGGATCCTCAGACACCCGACTCATCCTG	
prad-*dg_pprI*ΔC R	CCTGCAGGTCGAATCGGATCCTGAGGTTCGCTCCGCCAG	
dg_pprI H46A F	CGCTCATCAGGCTGGCGGTATCCAGCCCCG	
dg_pprI H46A R	CGGGGCTGGATACCGCCAGCCTGATGAGCG	
dg_pprI F58A F	TCACCCATCGGCATAGCGGTGAGGGTCGCGTC	
dg_pprI F58A R	GACGCGACCCTCACCGCTATGCCGATGGGTGA	
dg_pprI R85A F	GTGAAGCGCTGGGCTTCCGGGCGTACCC	
dg_pprI R85A R	GGGTACGCCCGGAAGCCCAGCGCTTCAC	

	Sequence (5′→3′)	Label
dg_pprI R207A F	CCGCACTGGCCGCAACAGTAAGCGCCTTTTCG	
dg_pprI R207A R	CGAAAAGGCGCTTACTGTTGCGGCCAGTGCGG	
dg_pprI R267A F	AAAACTCACCAGCACCGCCTGACGCTCAGGAAAG	
dg_pprI R267A R	CTTTCCTGAGCGTCAGGCGGTGCTGGTGAGTTTT	
dg_pprI R220A F	GTGAAGTACAGCCTGGCCCCCGGCACGCTCATC	
dg_pprI R220A R	GATGAGCGTGCCGGGGGCCAGGCTGTACTTCAC	
dg_pprI R250A F	GTTACGTGCCCTTCGCTTCGGGGCGGCGGATG	
dg_pprI R250A R	CATCCGCCGCCCCGAAGCGAAGGGCACGTAAC	
dg_pprI S251A F	GTTACGTGCCCTTCCGTGCGGGGCGGCGGATGC	
dg_pprI S251A R	GCATCCGCCGCCCCGCACGGAAGGGCACGTAAC	
dg_pprI R250A/S251A F	GTTACGTGCCCTTCGCTGCGGGGCGGCGGATGC	
dg_pprI R250A/S251A R	GCATCCGCCGCCCCGCAGCGAAGGGCACGTAAC	
dg_pprI L22A F	CCGGAGACTGGAGCGGCGGCCCCGGCCAAGGCGC	
dg_pprI L22A R	GCGCCTTGGCCGGGGCCGCCGCTCCAGTCTCCGG	
dg_pprI K26A F	AGCGCTGGCCCCGGCCGCGGCGCGTATGCGGGAACT	
dg_pprI K26A R	AGTTCCCGCATACGCGCCGCGGCCGGGGCCAGCGCT	
dg_pprI L22A/K26A F	CCGGAGACTGGAGCGGCGGCCCCGGCCGCGGCGC	
dg_pprI L22A/K26A R	GCGCCGCGGCCGGGGCCGCCGCTCCAGTCTCCGG	
dg_pprI R117A F	CGCCTACGAGGGAGAGGCGTTGGAGCAGGTCATAG	
dg_pprI R117A R	CTATGACCTGCTCCAACGCCTCTCCCTCGTAGGCG	
dg_pprI 4A F	GCCCCCGCGGCCGCCGTGGTGCTGATCAACAGTC	
dg_pprI 4A R	GGCGGCCGCGGGGGCATAGGCTCCGTCACGGTCA	
dg_ddrO F	GTGCCGCGCGGCAGCCATATGAAACTGCACGAACGACTTCG	
dg_ddrO R	ACGGAGCTCGAATTCGGATCCTCAGCCCAGAATGCGCTTG	
dg_ddrO E116A F	GCTTGCCGCGCAGCGCGATGCGGGACCTC	
dg_ddrO E116A R	CCTGTCCCGCATCGCGCTGCGCGGCAAGC	
dg_ddrO L117A F	GCGCTTGCCGCGCGCCTCGATGCGGGAC	
dg_ddrO L117A R	GTCCCGCATCGAGGCGCGCGGCAAGCGC	
dg_ddrO R118A F	GGGCGCTTGCCGGCCAGCTCGATGCG	
dg_ddrO R118A R	CGCATCGAGCTGGCCGGCAAGCGCCC	
dg_ddrO G119A F	CCGCGGGCGCTTGGCGCGCAGCTCGATG	
dg_ddrO G119A R	CATCGAGCTGCGCGCCAAGCGCCCGCGG	
eY/CFP F	GTGCCGCGCGGCAGCCATATGGTGAGCAAGGGCGAGG	
eY/CFP R	CGAGCCACCGCCTCCTGAGCCACCGCCTCCCTTGTACAGCTCGTCC ATGCC	
dg_pprI-eY/CFP F	GCTCAGGAGGCGGTGGCTCGCCGGAGACTGGAGCGCTGG	
dg_pprI-eY/CFP R	CGGAGCTCGAATTCGGATCCTCAGACACCCGACTCATCCTGCTCGC	
dg_ddrO-eCFP F	GCTCAGGAGGCGGTGGGTGCGAGGCAACGCCGCAGCCGAG	

	Sequence (5′→3′)	Label
dg_ddrO-eCFP R	CGGAGCTCGAATTCGGATCCTCAGCCCAGAATGCGCTTGAGATGCA	
**RT-qPCR**		
dr_ddrO F	TTCGTGAATTGCGCAGCGAAC	
dr_ddrO R	GCAACTCGATGCGCGAGAG	
dr_1343 F	TTTGACGGCACCGTGGAGTAC	
dr_1343 R	GCATTGGAGATGATGTGGTGGTTG	
dr_recA F	GAGCAAACTCGACGTGCAGG	
dr_recA R	AGCAGTTCCATGATTTCGAGCG	
dr_uvrD F	GACCTCTTTACGCTGACCGAAGT	
dr_uvrD R	CTTCTGCGGCAAAGAGCGG	

Supplementary Table 4: ipTM+pTM of the five AlphaFold-Multimer models

ranked	ipTM+pTM
**1**	0.799
**2**	0.557
**3**	0.496
**4**	0.478
**5**	0.223

Supplementary Table 5: Summary of MD simulation systems in this study

Label	Solute	Species	Solvent	Temperature (K)	Time (ns)
PprI+ssDNA/ DdrO (ternary model)	PprI monomer, ssDNA and DdrO dimer complex (with one Mn^{2+} ion)	*D. geothermalis*	150 mM KCl	310	500

Supplementary Figures

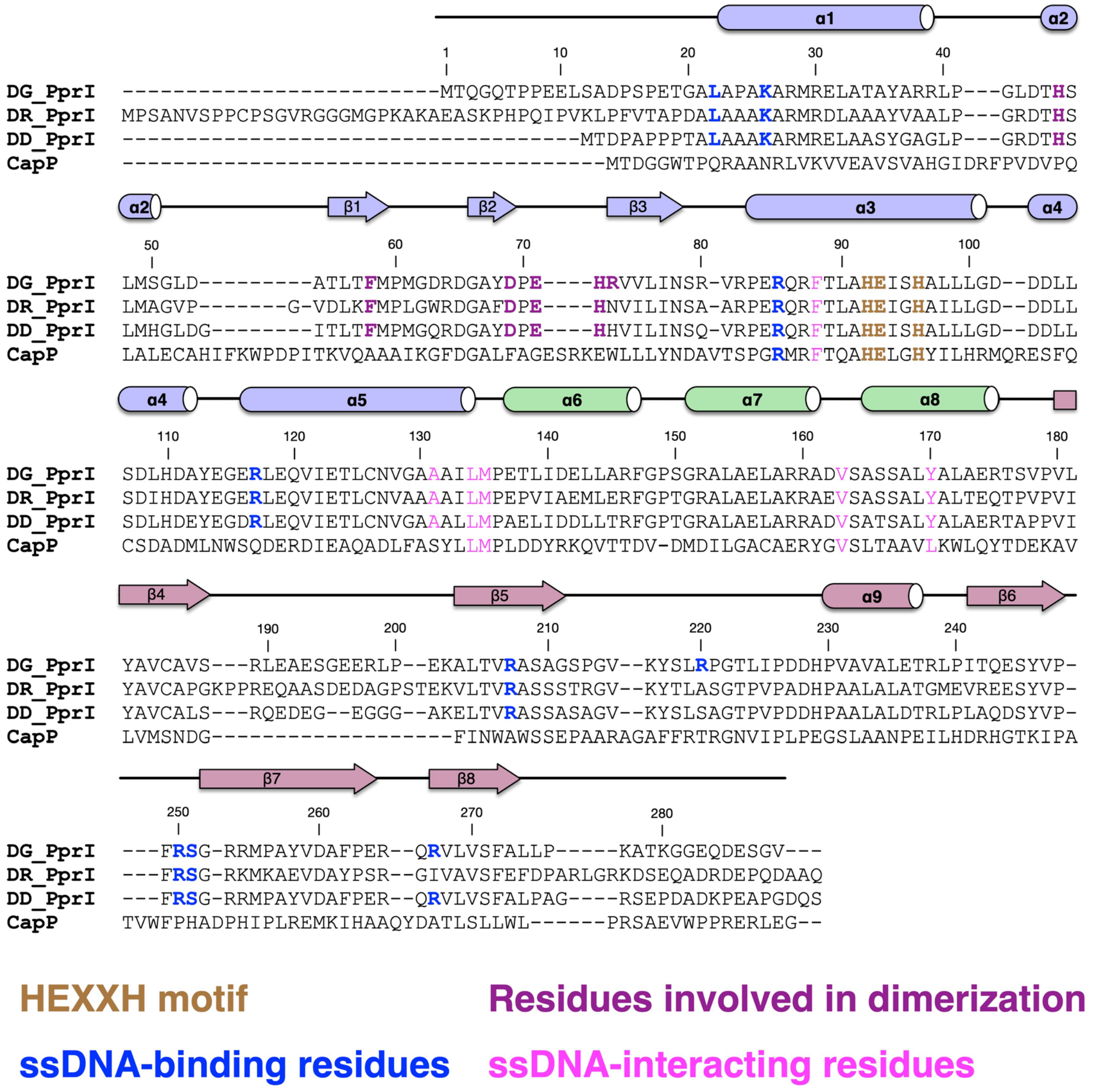

Figure S1. Structural based sequence alignments of the PprI from *D*einococcus species and CapP protein. PprI proteins from *D. geothermalis*, *D. radiodurans* and *D. deserti* are denoted by DG-PprI, DR-PprI and DD-PprI, respectively. CapP is from *Thauera sp.* K11 (PDB ID: 7T5T). Secondary structure of DG-PprI is shown above the protein sequences. Residues involved in HEXXH motif, ssDNA binding, dimer interfaces of DG-PprI are highlighted.

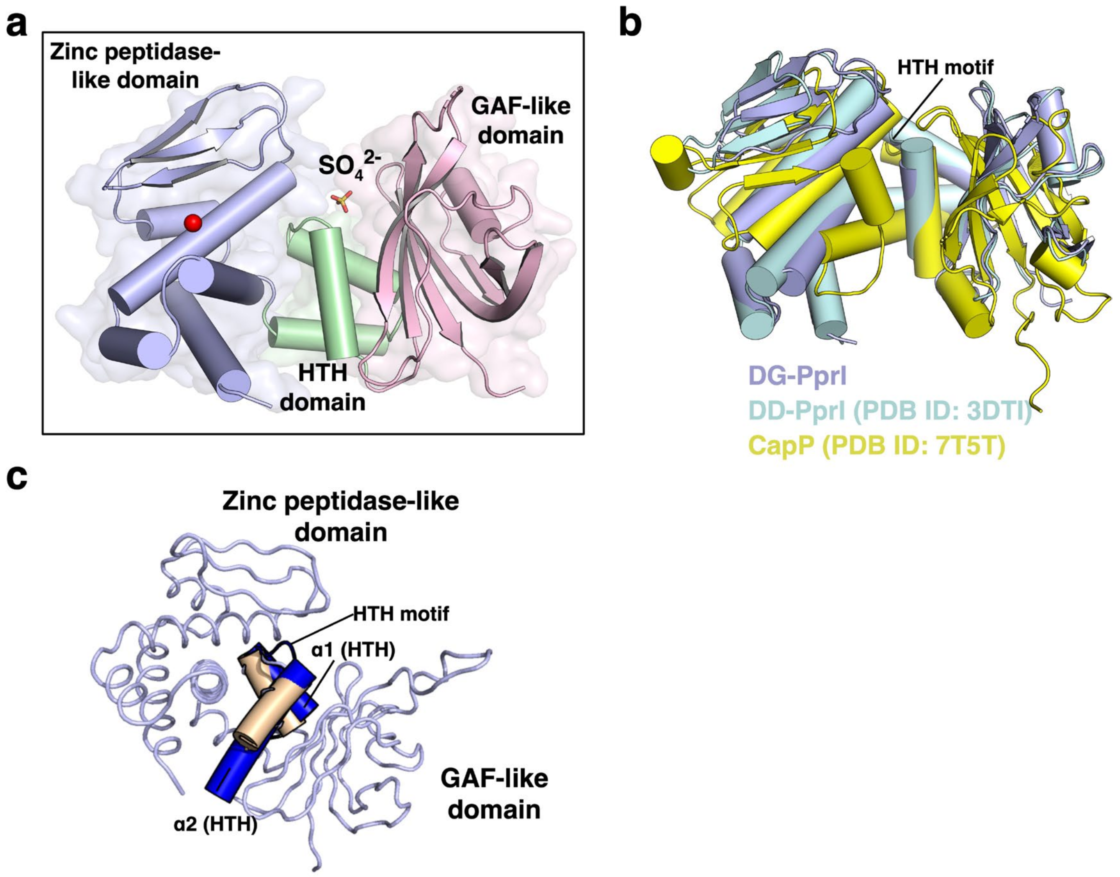

Figure S2. Comparison of PprI structures and HTH motifs. (a) Apo structure of the DG-PprI. Protein domains are labeled and shown in distinct colors, with the zinc peptidase-like domain, the helix-turn-helix domain, and the GAF-like domain in slate, green, and pink, respectively. The catalytic metal ion is shown as sphere and colored red. The sulfate radical is labeled and shown as sticks. **(b)** Superposition of DG-PprI (slate)， DD-PprI (palecyan, PDB ID: 3DTI) and CapP (yellow, PDB ID: 7T5T). **(c)** Structural alignment of HTH domains of DG-PprI (slate), LexA (blue, PDB ID: 3JSO) and DdrO (wheat, PDB ID: 6JQ1).

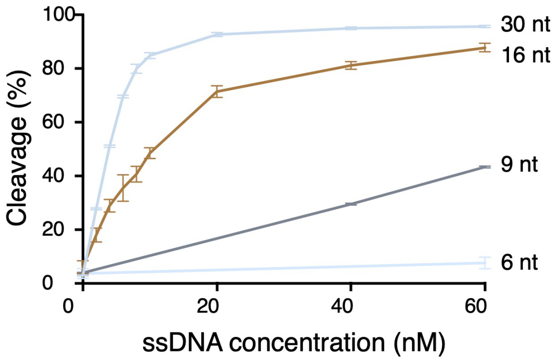

Figure S3. ssDNA activation assays containing various lengths (6-40nt) and concentrations (0-60 nM) of ssDNA using the same reaction conditions as in Fig 2e. Data represent the means of the three replicates, and the bars represent their standard deviations. Source data are provided as a Source Data file.

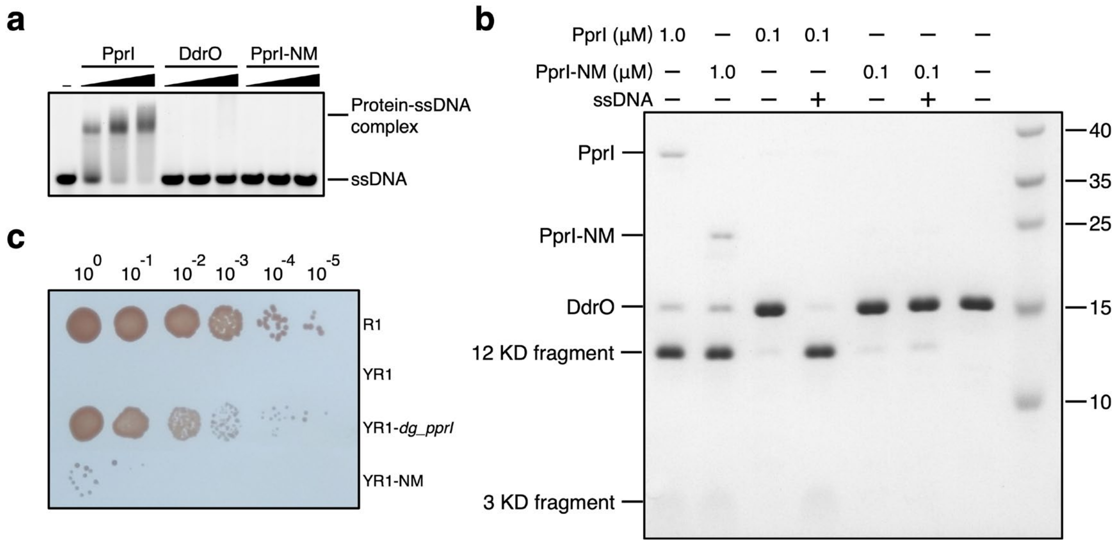

Figure S4. The GAF-like domain is required for the ssDNA activation. (a) The binding assay of wildtype PprI, DdrO and the GAF-like domain truncated PprI (PprI-NM) with ssDNA. The reaction conditions are the same as in Fig. 1d. **(b)** Cleavage and ssDNA activation assays of the GAF-like domain truncated PprI under the same reaction conditions as in Fig. 3d. **(c)** The phenotype assay of the GAF-like domain truncated strain. Wild-type strain (R1), *dr_pprI* knockout strain (YR1), and *dg_pprI* complementary strains (YR1-*dg_pprI* for the wild-type DG-PprI and YR1-NM for GAF-like domain truncated DG-PprI) were spotted on TGY medium following 4 kGy gamma radiation treatments. Source data are provided as a Source Data file.

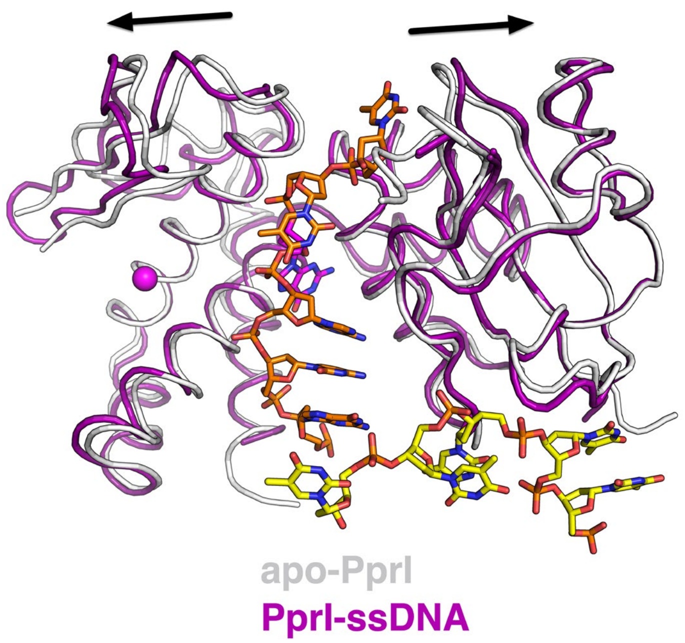

Figure S5. Superposition of apo PprI and PprI-ssDNA structures. The PprI-apo and PprI-ssDNA structures are colored in white and purple respectively. The arrowheads indicate a slightly enlarged sulphate-binding cavity.

ssDNA: 5’-TCATGAGCAG(10)TTT…TTTTTTT…

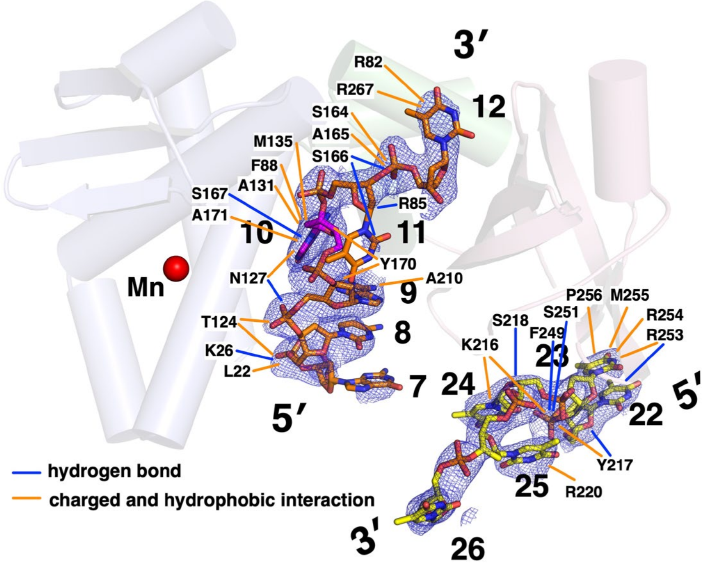

Figure S6. Schematic of the interactions between ssDNA and protein. Hydrogen bonds, charged and hydrophobic interaction were colored blue and orange, respectively. The electron density of two segments of ssDNA (5′-GCAGTT and 3′-TTTTT) is shown in blue with the refined 2Fo-Fc contoured at 1σ.

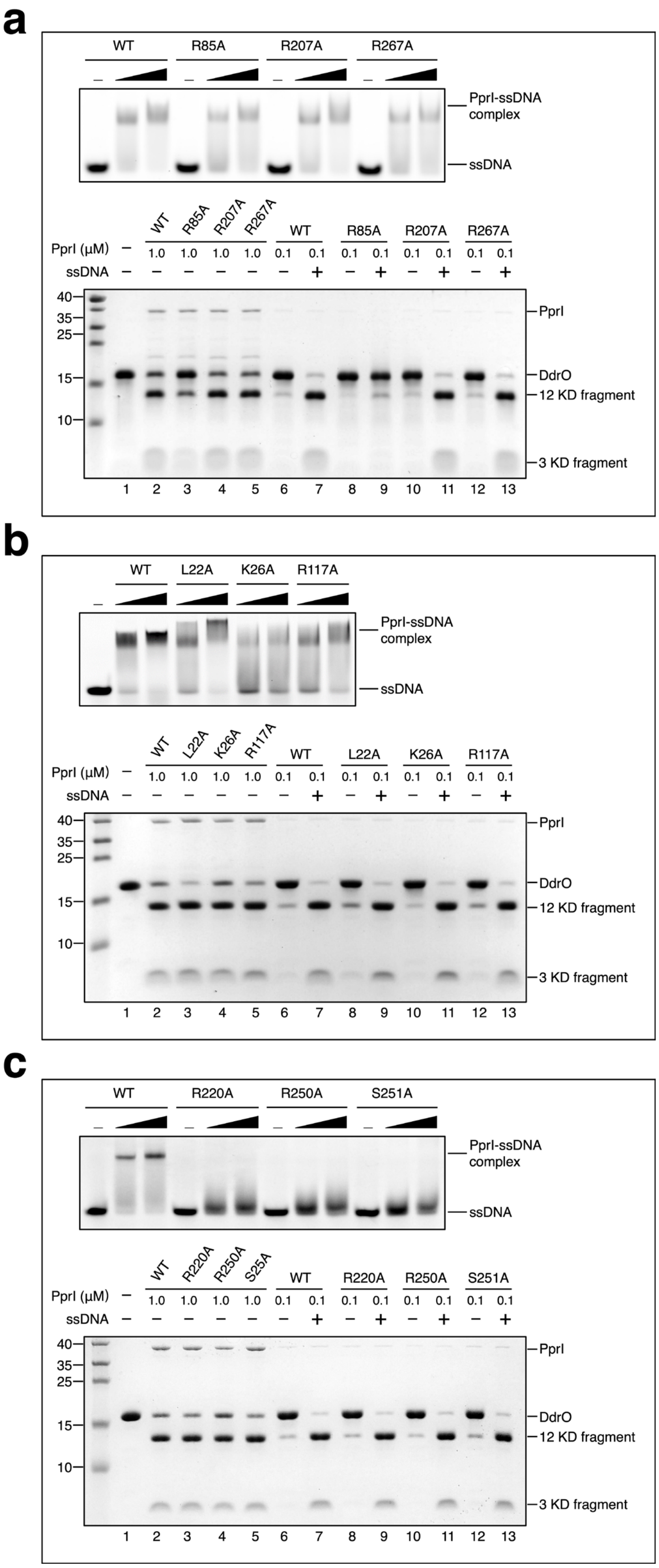

Figure S7. Cleavage and ssDNA activation assays of single mutants involved in patch1 **(a)**, patch2 **(b)**, and pactch3 **(c)** interactions. The reaction conditions are the same as in Fig. 3d. Source data are provided as a Source Data file.

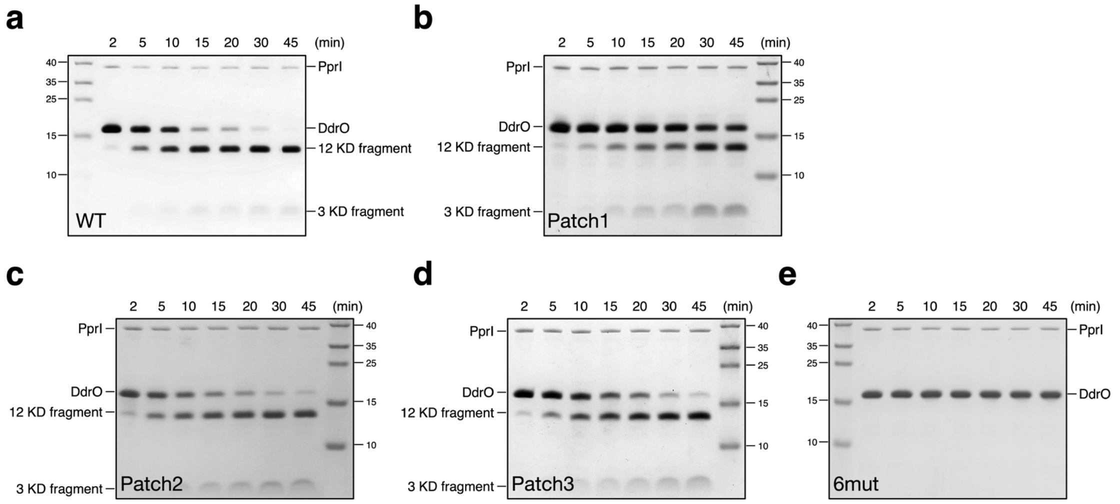

Figure S8. Timecourse (2, 5, 10, 15, 20, 30, 45 min) cleavage assays of the mutants. **(a)** wildtype PprI, **(b)** patch1 mutant, **(c)** patch2 mutant, **(d)** patch3 mutant, **(e)** 6mut. The reaction conditions are the same as in Fig. 3d. Source data are provided as a Source Data file.

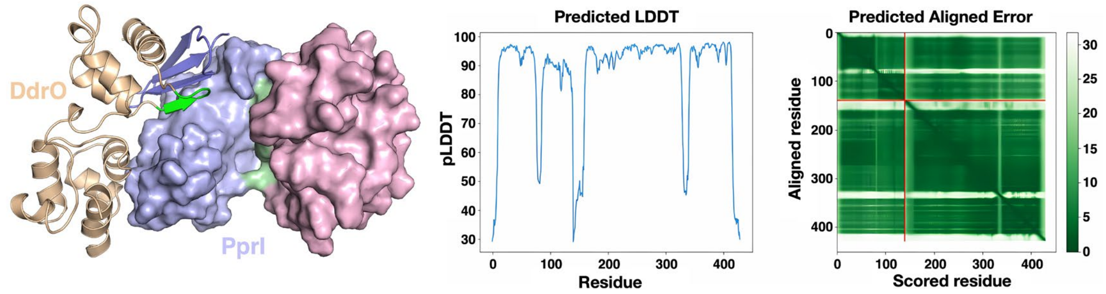

Figure S9. Left panel: PprI-DdrO complex model predicted using AlphaFold2 complex modeling tool. Right panel: The pLDDT (per-residue local distance difference test) and PAE (predicted aligned error) plots of the predicted model.

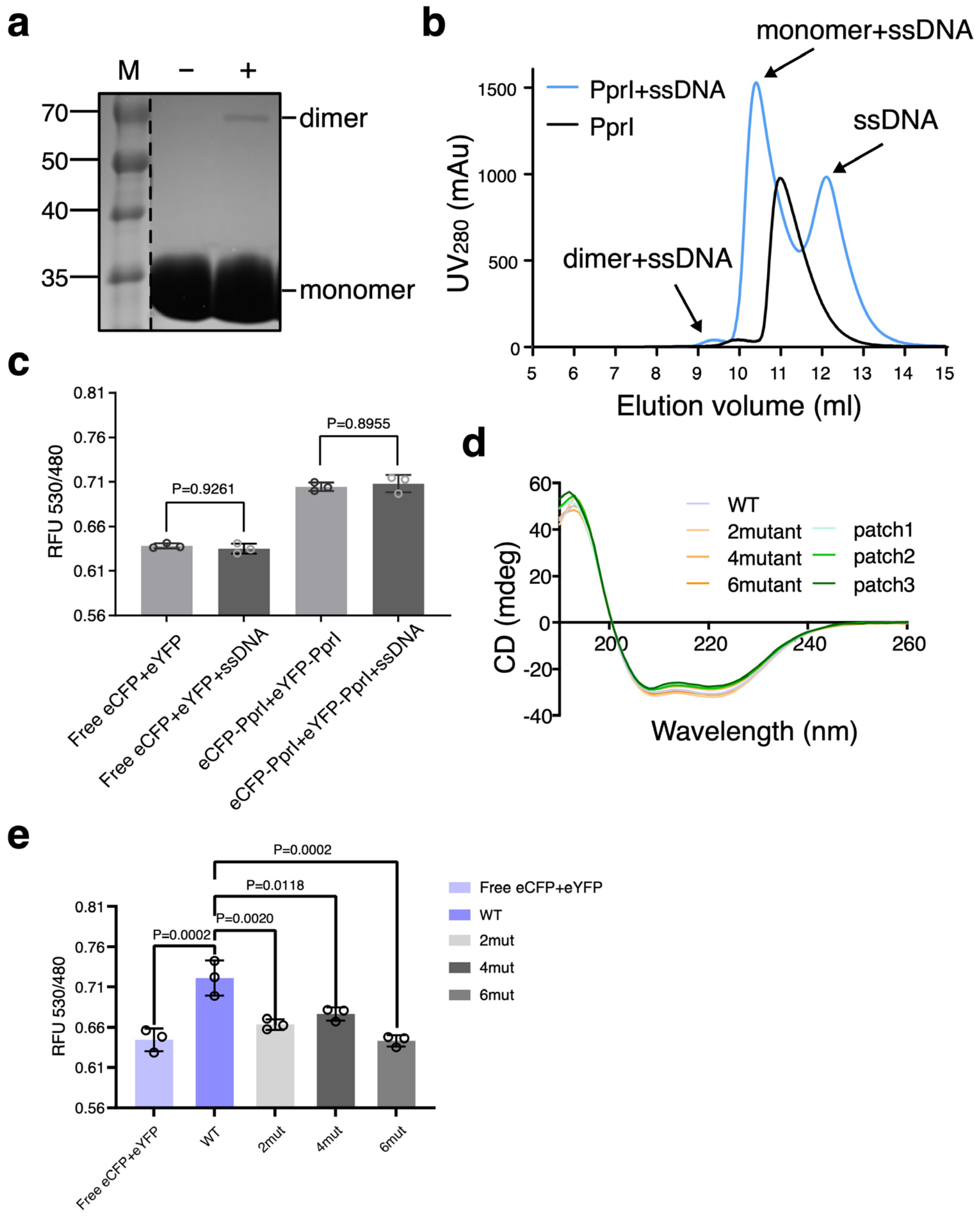

Figure S10. Monomer-dimer equilibrium of PprI. (a) Formaldehyde crosslink assay of PprI (1.7 mg/ml) by formaldehyde (25 mM). **(b)** Size exclusion chromatography of wild-type DG-PprI (4 mg/ml) incubated with 35nt ssDNA (0.7 mM) on Superdex 75 10/300 GL column. The peaks corresponding to monomeric or dimeric PprI proteins bounded to ssDNA are labeled. **(c)** FRET assays showing little effect of the addition of ssDNA on the PprI dimeric interactions. Data represent the means of the three replicates (bars represent standard deviations). **(d)** CD spectra of 0.2 mg/ml DG-PprI mutants in PB buffer. **(e)** FRET assays showing the effect of the interface mutation on the PprI dimeric interactions. Data represent mean ± SD of three independent experiments, compared with one-way ANOVA method followed by Tukey's post-hoc test. Source data are provided as a Source Data file.

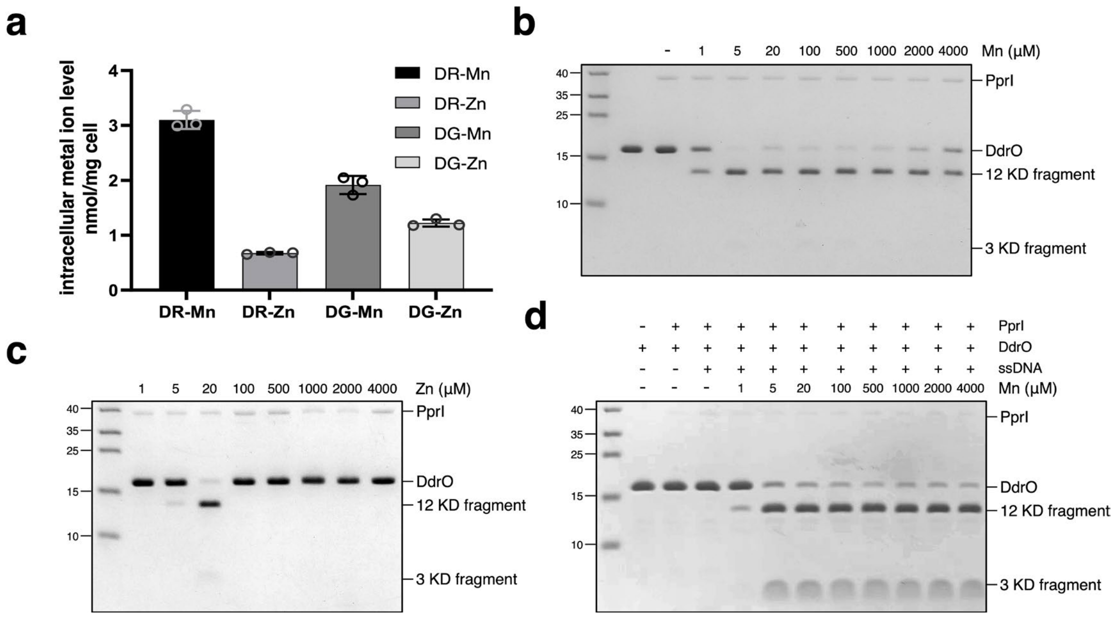

Figure S11. Different effects of zinc and manganese on the cleavage efficiency of DdrO by PprI in vitro, as well as the different concentrations of these two metals in the cell in vivo. (a) Intracellular concentrations of Mn and Zn of *D. geothermalis* and *D. radiodurans* using ICP-MS. Data represent mean ± SD of three independent experiments. **(b)** PprI cleave assays with different concentrations of manganese ion (1, 5, 20, 100, 500, 1000, 2000, 4000 μM). **(c)** PprI cleave assays in the presence of different concentrations of Zinc ion (1, 5, 20, 100, 500, 1000, 2000, 4000 μM). **(d)** PprI activation assays with different concentrations of manganese ion (1, 5, 20, 100, 500, 1000, 2000, 4000 μM). Source data are provided as a Source Data file.

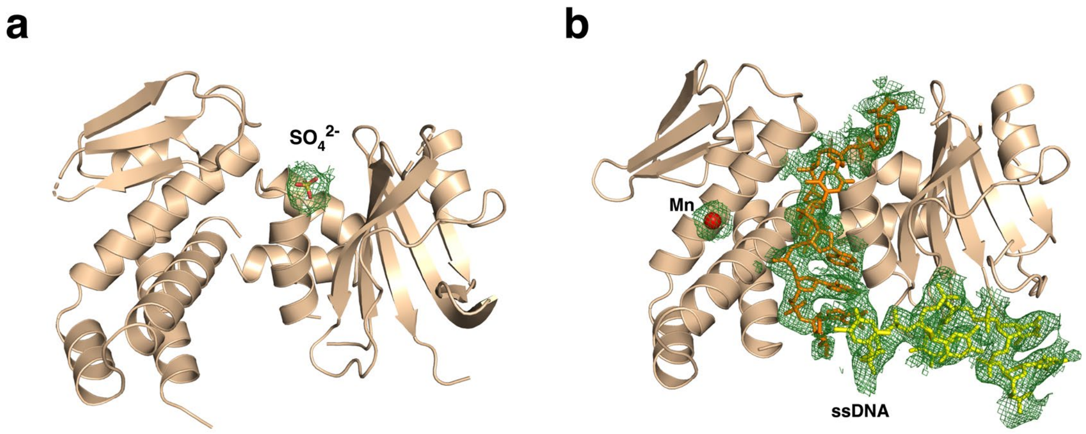

Figure S12. The omit map for sulfate of apo DG-PprI structure (8SLM) (a) and manganese ion and ssDNA of PprI-ssDNA complex (8SLN) (b). |(*Fo*)-(*Fc*)| difference density contoured at 1σ (green).
