# Supplementary material for: The Deinococcus protease PprI senses DNA damage by directly interacting with single-stranded DNA
Source: Nat Commun. 2024 Feb 29;15:1892. doi: 10.1038/s41467-024-46208-9 (PMC10904395; doi:10.1038/s41467-024-46208-9)
Supplement: Supplementary file 3 — Description of Additional Supplementary Files [file 41467_2024_46208_MOESM3_ESM.pdf]

## Description of Additional Supplementary Files:

**Supplementary Dataset 1:** The coordinates, maps and validation reports for crystal structures 8SLM (PprI-apo) and 8SLN (PprI-ssDNA complex).

**Supplementary Dataset 2:** The input set of coordinates for molecular dynamics simulations (input_model_before_MD.pdb).

**Supplementary Dataset 3:** The output set of coordinates for molecular dynamics simulations (output_model_after_MD.pdb).
